# Intermittent theta burst stimulation vs. high-frequency repetitive transcranial magnetic stimulation for post-stroke dysfunction: a Bayesian model-based network meta-analysis of RCTs

**DOI:** 10.1007/s10072-024-07918-6

**Published:** 2024-12-21

**Authors:** Yanbing Huang, Caihui Li, Rongda Cai, Tianlai Lin, Weiwen Chen

**Affiliations:** 1 Department of Critical Care Medicine, Quanzhou First Hospital Affiliated to Fujian Medical University, Quanzhou, Fujian Province 362000 China; 2 Quanzhou First Hospital Affiliated to Fujian Medical University, No.250 East Street, Licheng District, Quanzhou, Fujian Province 362000 China

**Keywords:** Post-stroke dysfunction, iTBS, HF-rTMS, Network meta-analysis

## Abstract

**Objective:**

This research aims to comprehensively assess the efficacy of intermittent theta-burst stimulation (iTBS) vs. high-frequency repetitive transcranial magnetic stimulation (HF-rTMS) in post-stroke dysfunction.

**Materials and methods:**

Until January 2024, extensive electronic database searches were conducted (PubMed, Embase, Cochrane Library, Web of Science, etc.). Fugl-Meyer Assessment for Upper Extremities (FMA-UE) was used to assess upper limb (UL) dysfunction; post-stroke dysphagia (PSD) was identified by Standardized Swallowing Assessment (SSA), Fiberoptic Endoscopic Dysphagia Severity Scale (FEDSS), and Penetration/Aspiration Scale (PAS). Results were analyzed by network meta-analysis (NMA), and the mean difference (MD) and 95% confidence intervals (95% CI) were also reported. We conducted a descriptive analysis due to the inability to synthesize data on post-stroke cognitive impairment (PSCI).

**Results:**

19 studies were included for NMA analysis. For UL disorder, the efficacy of treatments was ranked as HF-rTMS [MD (95%CI):3.00 (1.69,4.31)], iTBS [MD (95%CI): 2.16 (0.84, 3.50)], and sham stimulation (reference). For PSD, the efficacy of treatment to reduce scores of FEDSS or SSA were iTBS [FEDSS, MD (95%CI): -0.80 (-1.13, -0.47); SSA, MD (95%CI): -3.37 (-4.36, -2.38)], HF-rTMS [FEDSS, MD (95%CI): -0.43 (-0.76, -0.10); SSA, MD (95%CI): -2.62 (-3.91, -1.35)], and sham stimulation(reference). Descriptive analysis of PSCI found that both iTBS and HF-rTMS were effective in improving PSCI.

**Conclusions:**

HF-rTMS demonstrates superior efficacy in UL dysfunction, while iTBS is more effective in PSD. Clinicians should carefully evaluate the type and severity of post-stroke dysfunction in each patient to select the most appropriate treatment.

**Supplementary Information:**

The online version contains supplementary material available at 10.1007/s10072-024-07918-6.

## Introduction

According to the World Stroke Organization, stroke is one of the main causes of death and disability in the globe, with an estimated 12.2 million stroke events occurring annually [1]. A study found that over 60% of stroke survivors have moderate or severe functional impairment [2]. Post-stroke dysfunction includes increased patient incapacity such as motor impairment, and cognitive ability [3, 4], which seriously affects the quality of life of patients. Upper limb (UL) dysfunction is estimated to occur in 55–75% of post-stroke patients [5, 6], which may lead to a health-related reduction in quality of life. In addition, post-stroke dysphagia (PSD) is also a common complication of stroke [7] and previous studies have reported an association with an increased risk of acute-phase mortality, malnutrition, dehydration, and increased length of hospital stay, although conventional treatment remains limited [8].

The noninvasive brain stimulation (NIBS) technique, a method of promoting functional recovery and improvement by stimulating neuronal activity in the brain, has been shown to improve stroke [9]. After stroke, patients often experience decreased cortical excitability, dysfunction, vascular edema, and interhemispheric imbalance, which together lead to severe motor dysfunction [10]. Non-invasive brain stimulation (NIBS) promotes the recovery of motor function by regulating cortical excitability and reducing interhemispheric inhibition [10]. In addition, NIBS may contribute by modulating synaptic plasticity in brain neurons and enhancing the functional connectivity of neural networks [11]. High-frequency repetitive transcranial magnetic stimulation (HF-rTMS) is a technique that delivers high-frequency magnetic pulses to specific areas of the brain to increase the cortical excitability of regions targeted by placing electromagnetic coils on the scalp [12]. HF-rTMS is widely used in the treatment of neurological disorders such as depression, Parkinson’s disease, and sequelae of a stroke [13–15]. Intermittent theta-burst stimulation (iTBS) appears to be a novel rTMS modality that delivers pulses that imitate theta rhythms in the brain [16]. Currently, iTBS is also frequently used for many psychological disorders [17, 18].

A study in which 20 chronic stroke patients received two different stimulation conditions: single iTBS or sham stimulation of the ipsilateral M1, found that iTBS promoted intracortical excitability in chronic stroke patients [19]. Another meta-analysis showed that HF-rTMS, low-frequency rTMS (LF-rTMS), and iTBS all had beneficial effects on overall cognitive functioning in stroke patients [20]. Currently, although several studies have investigated the effects of the two approaches separately, there are relatively few direct comparative studies on iTBS and HF-rTMS for the treatment of post-stroke dysfunction, and it is not clear which stimulation method is more effective for functional recovery in stroke sequelae. A critical question about which treatment is most effective cannot be answered using traditional meta-analytic methods [21]. Moreover, traditional meta-analyses do not allow for comprehensive analyses of trials investigating multiple treatment groups in a study because they compare only two treatments at a time.

Therefore, randomized controlled trials (RCTs) on iTBS, HF-rTMS, sham stimulation, and basic stroke treatment on post-stroke dysfunction have been widely collected and based on which network meta-analysis (NMA) was constructed. This study aimed to assess the comparative efficacy of these therapies on UL dysfunction, PSD, and post-stroke cognitive impairment (PSCI) to establish a hierarchy of these interventions.

## Materials and methods

A comprehensive literature review was executed following the standards set by Preferred Reporting Items for Systematic Reviews and Meta-Analysis (PRISMA) [22] and the Cochrane Handbook for Systematic Reviews of Interventions [23].

### Search strategy

The search for data was extended until January 15, 2024, encompassing electronic databases including PubMed, Embase, the Cochrane Library, Web of Science, the China National Knowledge Infrastructure (CNKI), the China Science and Technology Journal Database (VIP), and Wanfang Data. A complete list of the search keywords and search terms can be found in Table Supplementary 1.

### Eligibility criteria

The PICOS tool served as the foundation for the search strategy: (P) Population: adult patients (age ≥ 18 years old) diagnosed with stroke through computed tomography or magnetic resonance imaging (MRI) and without conscious impairment; (I) Intervention: iTBS or HF-rTMS; (C) Comparator: basic stroke treatment, sham stimulation, sham stimulation + basic stroke treatment; (O) Outcomes: outcomes included UL dysfunction, PSD, and PSCI; (S) Study design: RCTs. All of the above interventions had a duration of at least one week, excluding interventions with a duration of less than one week.

The following scales were used for outcomes assessments: UL dysfunction was assessed by Fugl-Meyer Assessment for Upper Extremities (FMA-UE); PSD was identified by Standardized Swallowing Assessment (SSA), Fiberoptic Endoscopic Dysphagia Severity Scale (FEDSS), and Penetration/Aspiration Scale (PAS); assessments of PSCI comprised Mini-mental State Examination (MMSE) and Montreal Cognitive Assessment (MoCA).

Articles with the following characteristics were excluded: (1) animal experience; (2) retracted studies; (3) reviews, meta-analyses, errata, case reports, conference abstracts, editorial materials, letters, notes, and books; (4) non-English literature; (5) studies that do not match the topic.

### Data extraction

Primarily, EndNote X9 literature management software was used to remove duplicate records. An initial screening of the literature was performed by two reviewers judiciously through the examination of titles and abstracts. Following that, the full texts of the remaining articles were downloaded and further evaluated. Any discrepancies were addressed through discussion between the reviewers and consultation with the superintendent. After screening potentially eligible articles, two reviewers independently extracted and collected data from the studies. A pre-design extraction table was utilized to extract relevant information, encompassing the subsequent elements: the characteristics of trials [authors, year of publication, and country of enrolment), the characteristics of the patient (age, type of stroke, affected hemisphere of stroke patients, site of lesion, and presence of comorbidities (hypertension/diabetes/dyslipidemia, etc.)], intervention characteristics (type of intervention, period of intervention, duration of follow up), and outcome of concern (UL dysfunction, PSD, and PSCI).

### Risk of bias assessment

The risk of bias was assessed strictly under the guidelines of the Cochrane Handbook [24] which scored the included studies on the following: Selection bias, implementation bias, measurement bias, follow-up bias, reporting bias, and other biases.

### Certainty of the evidence

The Grading of Recommendations Assessment, Development, and Evaluation (GRADE) technique was used to assess the quality of the NMA evidence. The quality of evidence was evaluated across five dimensions: risk of bias, inconsistency, indirectness, imprecision, and publication bias. GRADE evidence was scored on four levels: high, moderate, low, and very low. The GRADE grading scale for this investigation was generated using the GRADEpro GDT online version [25].

### Statistical analysis

The statistical analysis was conducted using the Gemtc 1.0.1 package in R Studio, implementing a Bayesian Markov chain Monte Carlo (MCMC) framework for NMA. Four MCMC chains were used for simulation. The number of initial iterations is set at 20,000, followed by an additional 50,000 iterations, with a step length of 1. Depending on the level of heterogeneity, either a random-effects or fixed-effects model was selected for analysis. Consistency, a crucial assumption of NMAs, was evaluated by comparing the deviance information criterion (DIC) between consistency and inconsistency models. A smaller DIC value indicates better model fit, and a difference of less than 5 suggests acceptable consistency.

Network diagrams were generated using Stata 15 to visually represent the relationships among different interventions. For UL dysfunction, and swallowing dysfunction, network plots were created and the mean differences (MD) along with 95% confidence intervals (95% CIs) for all comparisons were reported. Forest plots were used to present the MD values and 95%CIs of comparisons. Additionally, rank probability plots were generated to compare the rankings of iTBS and HF-rTMS, with the probabilities of each intervention being represented on the x-axis. Subgroup analysis was performed according to stimulated areas in the assessment of NIBS and SSA. Sensitivity analyses were also conducted to assess the robustness of the results.

## Results

### Characteristics of the eligible studies

**Fig. 1:**
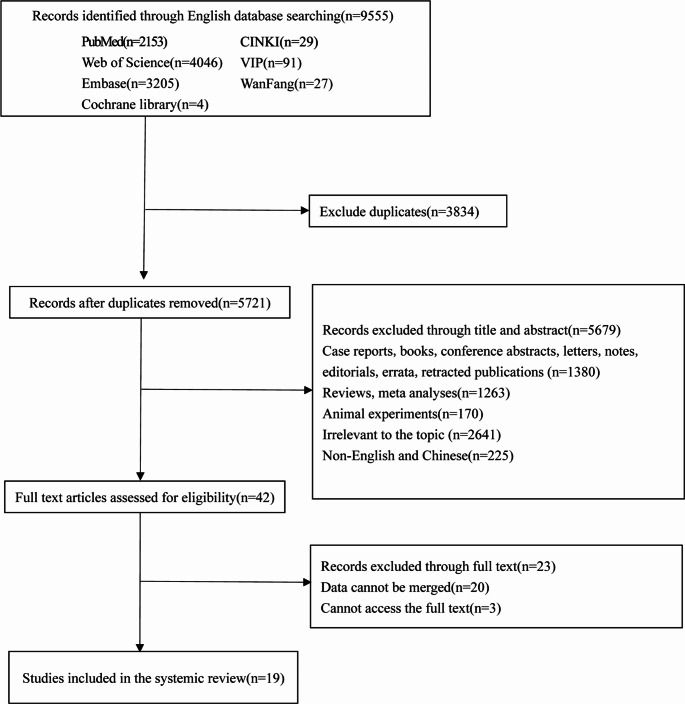
The flowchart of the study

In the beginning, a total of 9,555 documents were searched according to the search strategy. After deleting 3,834 article duplicates, 5,721 articles were retained for titles and abstract screening. A full-text review of 42 articles was conducted, resulting in 19 eligible studies [26–44] being used for systematic review and meta-analysis. More details of the process of inclusion are presented in Fig. 1.

Table 1 reveals the characteristics of the study with a total of 1,085 participants. All of the studies were published between 2017 and 2023. Of the 19 trials, most of the studies focused on comparisons between iTBS or HF-rTMS and sham stimulation. Furthermore, the majority of the studies’ individuals had subacute strokes, and their ages were largely comparable, ranging from 48.6 to 69.5.

**Table 1:** The characteristics of the included studies

Author	Year	Stroke stage	Sample	Treatment	Stimulate brain regions	Age (year)	M/F	Ischemic/ Hemorrhage	Affected hemisphere (Right/Left)	Diseased region (Subcortex/Cortex)	Complication	Intervention period (w)	Follow-up (w)	Outcome
Chen [27]	2019	Chronic	22	iTBS	Ipsilesional M1	52.90 ± 11.10	7/4	2/9	5/6	NA	NA	2	NA	FMA-UE
				Sham iTBS	Ipsilesional M1	52.60 ± 8.30	7/4	3/8	2/9	NA				
Guan [28]	2017	Acute	42	HF-rTMS	Ipsilesional M1	59.70 ± 6.80	16/5	NA	10/11	21/0	NA	2	4, 12, 24, 48	FMA-UE
				Sham rTMS	Ipsilesional M1	57.40 ± 14.00	14/7	NA	9/12	21/0				
Kim [29]	2020	Subacute	40	HF-rTMS	Lesional M1	62.70 ± 14.20	15/5	NA	12/8	7/13	NA	8.2 ± 5.3	13	FMA-UE
				Sham HF-rTMS	Ipsilesional M1	64.00 ± 13.40	10/10	NA	13/7	5/15		7.9 ± 5.5		
Zhang [44]	2022	Chronic	28	iTBS	Ipsilesional M1	58.21 ± 9.00	9/5	6/8	6/8	11/3	Yes	3	2	FMA-UE
				Shami TBS	Ipsilesional M1	64.00 ± 5.40	7/7	10/4	7/7	10/4				
Li [30]	2022	Subacute	58	iTBS	Ipsilesional M1	69.50 (60.00, 78.00)	16/12	18/10	12/16	14/14	NA	2	NA	MMSE
				Sham iTBS	Ipsilesional M1	66.00 (53.00, 75.00)	18/12	14/16	6/24	18/12				
Liu [33]	2020	Chronic	62	TMS	Non-affected prefrontal cortex and dorsolateral area	58.55 ± 6.24	10/19	20/9	18/11	NA	NA	4	NA	MMSE
				Sham stimulation	Non-affected prefrontal cortex and dorsolateral area	57.69 ± 7.25	16/13	15/14	15/14	NA				
Zhang [43]	2019	Subacute	60	HF-rTMS	Non-affected prefrontal cortex and dorsolateral area	58.44 ± 16.6	20/10	20/10	18/12	NA	NA	4	NA	MoCA
				Sham stimulation	Non-affected prefrontal cortex and dorsolateral area	55.11 ± 18.03	18/12	18/12	18/12	NA				
Chen [26]	2022	Chronic	82	HF-rTMS	NA	64.28 ± 3.16	25/16	NA	NA	NA	NA	4	NA	MMSE
				Basic stroke treatment	NA	64.12 ± 3.21	23/18	NA	NA	NA				
Yin [42]	2018	Subacute	25	HF-rTMS	Mylohyoid motor cortex on both cerebral hemispheres	58.58 ± 11.98	11/1	10/2	NA	NA	NA	4	NA	MoCA
				Sham stimulation	Mylohyoid motor cortex on both cerebral hemispheres	60.15 ± 10.29	12/1	10/3	NA	NA				
Liu [31]	2022	Acute	49	HF-rTMS	Affected side suprahyoid cortex	67.61 ± 11.71	17/6	19/4	8/8	NA	Yes	2	2	SSA/FEDSS/PAS
				Sham rTMS	Affected side suprahyoid cortex	67.73 ± 9.97	20/6	21/5	10/13	NA				
Rao [36]	2022	All	70	iTBS	Affected prefrontal cortex and dorsolateral cortex	63.42 ± 10.35	22/11	15/18	18/14	20/5	Yes	2	2	SSA/FEDSS/PAS
				Shame iTBS	Affected prefrontal cortex and dorsolateral cortex	65.90 ± 11.42	24/7	19/12	12/12	11/5				
Tai [37]	2023	Subacute	90	TBS	Affected side suprahyoid cortex	58.40 ± 14.10	19/11	16/14	NA	NA	NA	4	2	SSA
				Sham TBS	Affected side suprahyoid cortex	57.07 ± 16.87	10/5	8/7	NA	NA				
Xie [40]	2022	Subacute	47	iTBS	Affected side suprahyoid cortex	67.50 ± 10.60	16/8	6/18	14/10	NA	NA	2	2	SSA/FEDSS
				HF-rTMS	Affected side suprahyoid cortex	64.80 ± 11.30	18/5	7/16	11/12	NA				
Liu [34]	2021	Subacute	89	iTBS	Affected side suprahyoid cortex	65.00 ± 9.93	16/8	11/13	NA	NA	Yes	2	2	SSA/PAS
				HF-rTMS	Affected side suprahyoid cortex	63.70 ± 10.05	17/13	22/8	NA	NA				
				Sham stimulation	Affected side suprahyoid cortex	63.43 ± 11.01	22/13	24/11	NA	NA				
Liu [32]	2022	Chronic	76	HF-rTMS	Affected side suprahyoid cortex	63.37 ± 3.82	21/17	21/17	23/15	16/11	Yes	4	NA	SSA/PAS
				Basic stroke treatment	Affected side suprahyoid cortex	62.62 ± 3.54	25/13	19/19	20/18	18/8				
Yan [41]	2019	Subacute	45	HF-rTMS	Mylohyoid motor cortex in both cerebral hemispheres	48.60 ± 6.97	10/5	6/9	6/9	NA	NA	4	NA	SSA
				Sham stimulation	Mylohyoid motor cortex in both cerebral hemispheres	51.27 ± 6.32	11/4	3/12	7/8	NA				
Wu [38]	2023	Acute	70	HF-rTMS	Non-lesional cerebellar pharyngeal motor area	63.28 ± 3.22	27/8	NA	NA	NA	NA	3	NA	SSA
				Basic stroke treatment	Non-lesional cerebellar pharyngeal motor area	63.18 ± 3.24	25/10	NA	NA	NA				
Xie [39]	2023	Subacute	70	HF-rTMS	Affected prefrontal cortex and dorsolateral cortex	65.82 ± 3.47	21/14	NA	NA	NA	NA	12	NA	SSA
				Basic stroke treatment	Affected prefrontal cortex and dorsolateral cortex	65.79 ± 3.35	20/15	NA	NA	NA				
Pei [35]	2022	Subacute	60	iTBS	Left dorsolateral prefrontal area M1	64.90 ± 5.46	18/13	26/5	NA	NA	NA	4	NA	MMSE/MOCA
				Sham stimulation	Left dorsolateral prefrontal area M1	66.93 ± 6.55	22/7	23/6	NA	NA				

RCT, Random control trail; iTBS, Intermittent theta-burst stimulation; HF-rTMS, High-frequency repetitive transcranial magnetic stimulation; TMS, Transcranial magnetic stimulation; M, Male; F, Female; NA, Not applicable; w, Week; FMA-UE, Fugl-Meyer assessment for upper extremities; MMSE, Minimum mental state examination; MoCA, Montreal cognitive assessment; SSA, Standard swallowing function evaluation scale; FEDSS, Fiberoptic endoscopic dysphagia severity scale; PAS, Penetration/aspiration scale. M1, Primary motor cortex

### Quality evaluation and research bias

As illustrated in Figure Supplementary 1, random sequence generation, and other biases were assessed as having a lower risk of bias, whereas incomplete outcome data was considered to have a higher risk of bias. Several articles [28, 30, 33, 40] did not exhibit a risk of bias entries by summarizing the risk of bias for each incorporated article (Figure Supplementary 2). In general, the studies included were considered to have an acceptable risk of bias.

By applying GRADE to assess the quality of the evidence, FEDSS, FMA-UE, PAS, and SSA were found to have relatively low-quality evidence (Figure Supplementary 3).

### Comparison of the efficacy of three intervention treatments for UL dysfunction after stroke

**Fig. 2:**
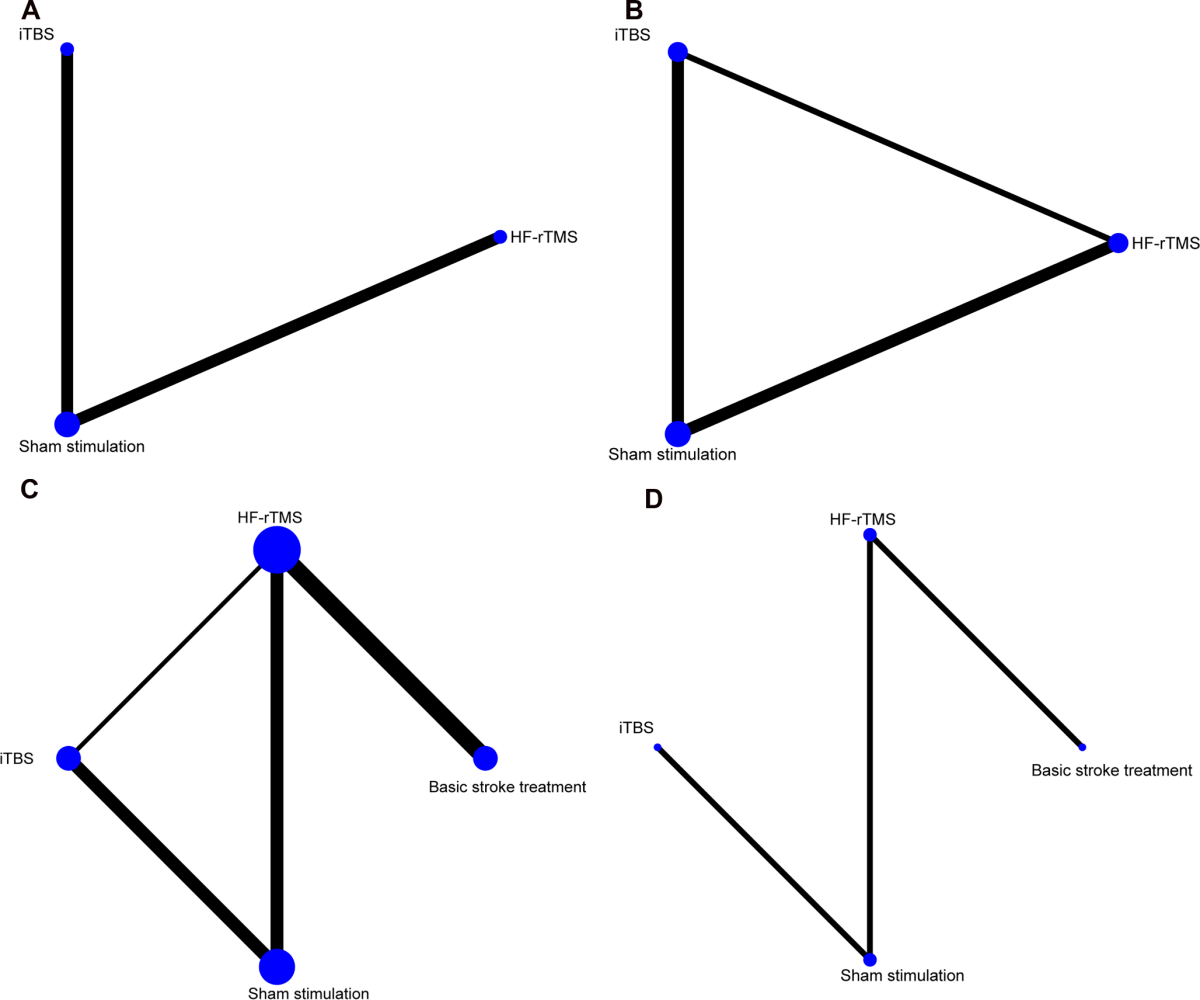
Geometry of the network. The node size indicates the number of participants in the intervention, while the edge thickness reflects the number of studies for each treatment comparison. (**A**) FMA-UE; (**B**) FEDSS; (**C**)SSA; (**D**) PAS. FMA-UE, Fugl-Meyer Assessment for Upper Extremities; FEDSS, Fiberoptic Endoscopic Dysphagia Severity Scale; SSA, Standardized Swallowing Assessment; PAS, Penetration/Aspiration Scale

**Fig. 3:**
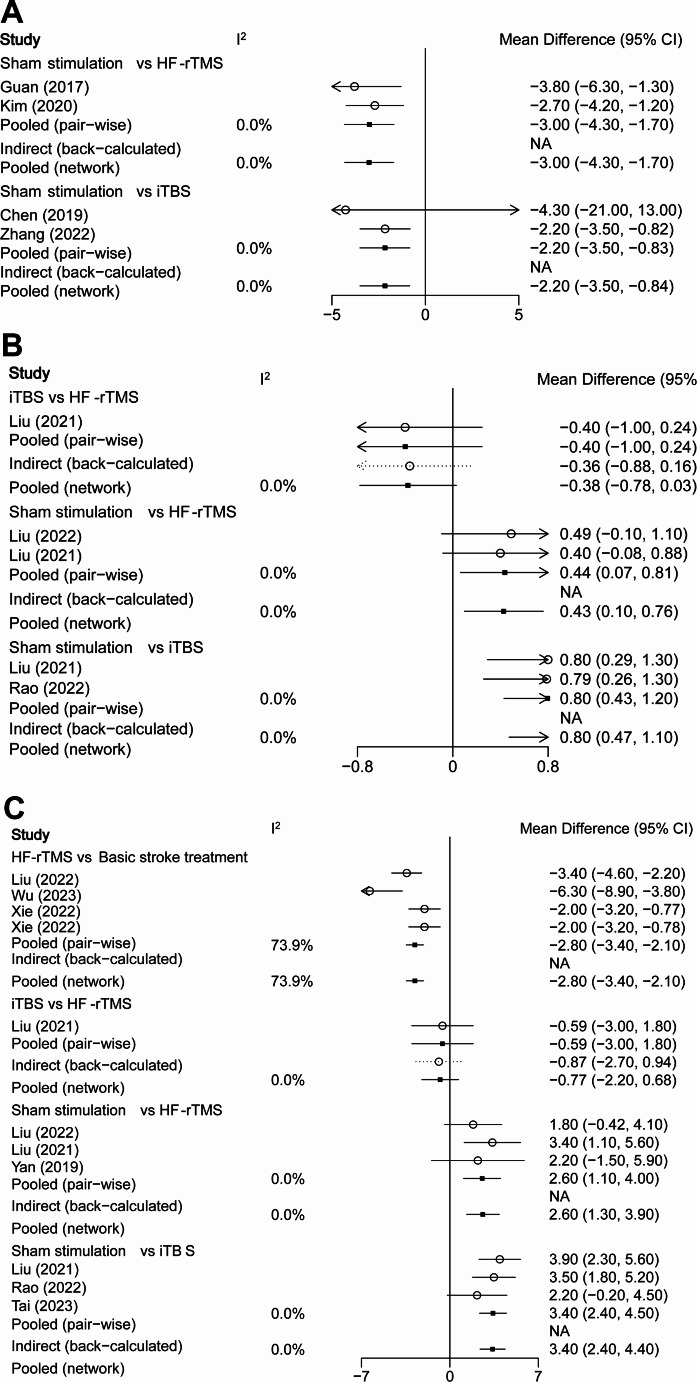
Meta-analysis for outcomes. (**A**) FMA-UE; (**B**) FEDSS; (**C**) SSA. FMA-UE, Fugl-Meyer Assessment for Upper Extremities; FEDSS, Fiberoptic Endoscopic Dysphagia Severity Scale; SSA, Standardized Swallowing Assessment

There were 4 RCTs included for analysis, comprising a total of 132 patients. As can be seen from Fig. 2A, the current study only provided a direct comparison between sham stimulation and iTBS or HF-rTMS. The connecting lines between sham stimulation and iTBS/HF-rTMS were of equal thickness, suggesting that the number of intervention studies was comparable. Additionally, the larger circle of sham stimulation indicated a larger sample size. Figure 3A presents the values of MD (95% CI) for sham stimulation vs. HF-rTMS and sham stimulation vs. iTBS were − 3.00 (95%CI = -4.30, -1.70) and − 2.20 (95%CI = -3.50, -0.84). There was no heterogeneity observed in the two groups (I^2^ = 0%). According to the rank probability plot (Figure Supplementary 4 A), the order of increased FMA-UE score was HF-rTMS > iTBS > sham stimulation. Thus, HF-rTMS might be the relatively effective treatment for UL dysfunction after stroke.

### Comparison of the efficacy of three intervention treatments for PSD

For FEDSS scores, 3 articles with a total of 202 patients were included. The studies of iTBS, HF-rTMS, and sham stimulation were directly compared to form a closed loop. Among them, the connecting line between sham stimulation and iTBS/HF-rTMS is thicker, indicating a larger number of studies (Fig. 2B). Figure 3B shows the MD values for sham stimulation vs. HF-rTMS and sham stimulation vs. iTBS were 0.43 (I^2^ = 0%, 95% CI = 0.10, 0.76) and 0.80 (I^2^ = 0%,95% CI = 0.47, 1.10). The league table (Table 2) demonstrates that sham stimulation vs. iTBS has the highest effect value (MD: 0.80, 95%CI = 0.47, 1.13), and sham stimulation vs. HF-rTMS had a medium effect value (MD: 0.43, 95%CI = 0.10, 0.76). According to Figure Supplementary 4B, the FEDSS scores ranked from highest to lowest were observed in the following order: sham stimulation, HF-rTMS, and iTBS. Higher FEDSS scores represented poorer swallowing function.

**Table 2:** Ranking of pairwise comparison of different treatments for post-stroke dysfunction

**FMA-UE**				
	HF-rTMS	iTBS	Sham stimulation	
HF-rTMS	HF-rTMS	-0.84 (-2.69, 1.02)	-3.00 (-4.31, -1.69)	
iTBS	0.84 (-1.02, 2.69)	iTBS	-2.16 (-3.50, -0.84)	
Sham stimulation	3.00 (1.69, 4.31)	2.16 (0.84, 3.50)	Sham stimulation	
**FEDSS**				
	HF-rTMS	iTBS	Sham stimulation	
HF-rTMS	HF-rTMS	-0.38 (-0.78, 0.03)	0.43 (0.10, 0.76)	
iTBS	0.38 (-0.03, 0.78)	iTBS	0.80 (0.47, 1.13)	
Sham stimulation	-0.43 (-0.76, -0.10)	-0.80 (-1.13, -0.47)	Sham stimulation	
**SSA**				
	Basic stroke treatment	HF-rTMS	iTBS	Sham stimulation
Basic stroke treatment	Basic stroke treatment	-2.78 (-3.45, -2.10)	-3.53 (-5.13, -1.93)	-0.16 (-1.59, 1.29)
HF-rTMS	2.78 (2.10, 3.45)	HF-rTMS	-0.76 (-2.20, 0.70)	2.62 (1.35, 3.91)
iTBS	3.53 (1.93, 5.13)	0.76 (-0.70, 2.20)	iTBS	3.37 (2.38, 4.36)
Sham stimulation	0.16 (-1.29, 1.59)	-2.62 (-3.91, -1.35)	-3.37 (-4.36, -2.38)	Sham stimulation
**PAS**				
	Basic stroke treatment	HF-rTMS	iTBS	Sham stimulation
Basic stroke treatment	Basic stroke treatment	-0.67 (-1.28, -0.06)	1.16 (-0.19, 2.52)	2.46 (1.37, 3.55)
HF-rTMS	0.67 (0.06, 1.28)	HF-rTMS	1.83 (0.62, 3.04)	3.13 (2.22, 4.04)
iTBS	-1.16 (-2.52, 0.19)	-1.83 (-3.04, -0.62)	iTBS	1.30 (0.49, 2.11)
Sham stimulation	-2.46 (-3.55, -1.37)	-3.13 (-4.04, -2.22)	-1.30 (-2.11, -0.49)	Sham stimulation

iTBS, Intermittent theta-burst stimulation; HF-rTMS, High-frequency transcranial repeated magnetic stimulation; FMA-UE, Fugl-Meyer assessment for upper extremities; SSA, Standard swallowing function evaluation scale; FEDSS, Fiberoptic endoscopic dysphagia severity scale; PAS, Penetration/aspiration scale

Nine studies were included in the analysis of SSA, involving 548 patients. Figure 2C indicates that iTBS, HF-rTMS, and sham stimulation were directly compared and studies conducted HF-rTMS exhibited a larger sample size. Figure 3C indicates that the value of MD (95% CI) for HF-rTMS vs. basic stroke treatment, sham stimulation vs. HF-rTMS, and sham stimulation vs. iTBS were − 2.80 (-3.40, -2.10), 2.60 (1.30, 3.90), and 3.40 (2.40, 4.40), respectively. The league table exhibits similar results (Table 2). Figure Supplementary 4 C concluded that the SSA scores in descending order are basic stroke treatment > sham stimulation > HF-rTMS > iTBS.

Three papers with 183 patients were included to assess the association of NIBS and PAS. Sham stimulation was compared directly with iTBS and HF-rTMS, and HF-rTMS was compared directly with sham stimulation and basic stroke treatment. The circles for HF-rTMS and sham stimulation were larger, indicating that their interventions were studied with larger sample sizes (Fig. 2D). Sham stimulation vs. HF-rTMS had high effect values, with an MD value of 3.13 (95% CI = 2.22, 4.04). Effect values were low for HF-rTMS vs. basic stroke treatment (MD: -0.67, 95%CI = -1.28, -0.06) (Table 2). In descending order, the PAS scores of different treatments were sham stimulation, iTBS, basic stroke treatment, and HF-rTMS (Figure Supplementary 4D).

### Comparison of the efficacy of three intervention treatments for PSCI

A total of 6 papers were included in the assessment of PSCI. In the comparison of HF-rTMS and the control group, HF-rTMS performed better in treating PSCI (*P* < 0.05) (Yin, 2018 [42]; Zhang, 2019 [43]; Liu, 2020 [33]; and Chen, 2022 [26]). The differences between post-intervention and pre-intervention were as follows: 5.75 ± 5.97 vs. 3.15 ± 5.44, 4.60 ± 4.08 vs. 1.36 ± 4.61, 3.07 ± 4.18 vs. 1.20 ± 3.37, and 8.37 ± 2.17 vs. 3.77 ± 2.28 for the HF-rTMS group and control groups, respectively. In literature conducted by Li et al. (2022) [30] and Pei et al. (2022) [35], iTBS was also found to be more effective than sham stimulation in treating PSCI (*P* < 0.05). The values of the median (interquartile range) between post-intervention and pre-intervention for the iTBS group were 20 (15, 23) vs. 12 (6, 17) in MoCA scores, while for the sham stimulation group, they were 13 (10, 20) vs. 9 (4, 15) [35]. Similarly, in the study conducted by Pei (2022) [30], the MMSE scores (post-intervention vs. pre-intervention) for the iTBS group were 17.00 (14.00, 24.00) vs. 12.50 (8.00, 19.00), and for the sham stimulation group, they were 14.00 (11.00, 18.75) vs. 11.00 (7.00, 15.00).

### Subgroup and sensitivity analyses

Because of the heterogeneity of SSA studies, subgroup analyses were performed according to the affected side or bilateral stimulation of NIBS. Table Supplementary 2 summarizes the NIBS priorities as iTBS, HF-rTMS, and sham stimulation. Excluding studies with influenced data (longest intervention duration, different stimulation sites) did not change the results (Table Supplementary 2).

## Discussion

To our understanding, this study is the first NMA to comprehensively assess the impact of iTBS, HF-rTMS, on multiple dysfunctions after stroke. This network analysis included 19 RCTs. The main findings of our study are as follows: (1) HF-rTMS therapy is superior to iTBS and sham stimulation in improving UL dysfunction; (2) iTBS seems to present a more positive effect than HF-rTMS and sham stimulation in improving patients’ swallowing function (FEDSS and SSA scales); (3) Either HF-rTMS or iTBS was effective in improving PSCI in stroke patients.

In our study, HF-rTMS was superior to iTBS or sham stimulation therapy in improving UL dysfunction. Previous research has demonstrated both iTBS and rTMS dramatically enhance the motor performance of UL compared to sham stimulation treatment [27, 28]. Damage to structural brain regions and their connections, as well as inhibition of the ipsilateral primary motor cortex (M1) and sensory cortex, disrupt the functional connectivity of motor networks after stroke and impair their flexibility [45]. Ipsilesional M1 was considered the key stimulating brain region of NIBS therapy. A meta-analysis also suggested that activation of the ipsilateral M1 and medial-premotor may be critical for motor recovery after stroke [46]. Du et al. [14] found an enhancement of cortical excitability in stroke patients receiving HF-rTMS, with a positive correlation between ipsilesional M1 activation and motor function. As a therapy of ipsilateral hemispheric excitatory repetitive NIBS, both iTBS and HF-rTMS can significantly promote the recovery of upper limb motor function and hand dexterity by enhancing the excitability of M1 on the affected side [47].

Dysphagia is another common stroke complication, and poor care may lead to serious consequences such as dehydration, pneumonia, and increased risk of acute-phase death [8]. Coincident with previous studies [48, 49], both iTBS and HF-rTMS significantly improved PSD. The stimulated brain regions of NIBS in improving PSD are mainly the motor cortex (especially the areas controlling the suprahyoid and mylohyoid muscles), cerebellum, and prefrontal cortex. Results of Lin et al. [50] showed that iTBS facilitated ipsilateral suprahyoid motor cortex excitability, and restored the balance of the bilateral hemispheres. Cerebellar NIBS can also elicit excitation of the swallowing motor cortex in the contralateral cerebral hemisphere through the cerebello-thalamo-cortical connections [51]. Additionally, cortical areas controlling oral and pharyngeal movements partially overlap, and iTBS may excite these overlapping areas, leading to co-enhancement [37]. This may explain the better performance of ITBS on the FEDSS and SSA scales observed in our study.

Both NIBC methods were found to be beneficial for PSCI in our study. Cholinergic pathways are often impaired in patients with mild cognitive impairment (MCI) [52]. Stimulation of the dorsolateral prefrontal cortex (DLPFC) may help restore this cholinergic innervation [53] and thereby improve cognitive function, which is also important in memory tasks in healthy people [54]. Another probable mechanism is the enhancement of long-term potentiation (LTP)-like plasticity [55]. LTP impairment has been proven to be associated with the level of cerebrospinal fluid (CSF) neurodegeneration [56], and plasticity impairment is central to cognition and recovery after neural injury [57]. A study by Koch et al. [56] demonstrated that stroke significantly reduced excitability and LTP in damaged areas, but rTMS improved LTP in patients with cognitive impairment. Both iTBS [58] and HF-rTMS [59] have been reported to enhance synaptic plasticity by promoting LTP to improve cognitive function.

In this research, we discovered that HF-rTMS and iTBS significantly improved patients’ UL dysfunction, PSD, and PSCI. Considering the risk of epilepsy induced by rTMS [60] and the relatively long treatment time, iTBS could be a potentially important modality for swallowing dysfunction that is treated. Our work serves as a valuable reference for future research and the development of therapeutic options, in addition to offering recommendations for the clinical practice of post-stroke dysfunction. It is crucial to recognize that our study has several limitations. Firstly, the follow-up periods in the included studies were short, with a lack of subsequent data collection, which limits the ability to assess the long-term efficacy and sustainability of the interventions. Additionally, the quality of the included studies varied, with some lacking rigorous methodological designs and sufficient follow-up periods, which could affect the reliability of the findings. Third, although iTBS showed better outcomes than HF-rTMS in treating PSD as measured by FEDSS and SSA, it was less effective than HF-rTMS in FMA-UE and PAS outcomes. Given the limited research directly comparing HF-rTMS and iTBS, this conclusion should be interpreted with caution. Finally, due to insufficient data on cognitive improvement, we were unable to perform an NMA analysis and to determine whether the improvement was specific to the stimulation site or network enhancement. In the future, more studies are needed to further verify the results and mechanisms.

## Conclusions

In summary, our study comprehensively assessed the efficacy of iTBS, HF-rTMS, and the control group (sham stimulation and basic stroke therapy) in improving post-stroke dysfunction. The available evidence suggests that HF-rTMS is more efficacious in improving UL deficits and iTBS is better in improving PSD. However, due to the variable quality of the included studies, this conclusion still needs to be further validated by large-sample, high-quality RCTs to provide a more reliable clinical basis.

## Electronic supplementary material

Below is the link to the electronic supplementary material.

Supplementary Material 1

Supplementary Material 2

Supplementary Material 3

Supplementary Material 4

Supplementary Material 5

Supplementary Material 6

## Data Availability

The datasets used and/or analysed during the current study were publicly available from the PubMed, https://pubmed.ncbi.nlm.nih.gov/; Embase, https://www.embase.com/; Cochrane Library, https://www.cochranelibrary.com/; Web of Science, https://www.webofscience.com/.
