# Supplementary figures and images for: Intermittent theta burst stimulation vs. high-frequency repetitive transcranial magnetic stimulation for post-stroke dysfunction: a Bayesian model-based network meta-analysis of RCTs
Source: Neurol Sci. 2024 Dec 21;46(4):1525–39. doi: 10.1007/s10072-024-07918-6 (PMC11919949; doi:10.1007/s10072-024-07918-6)

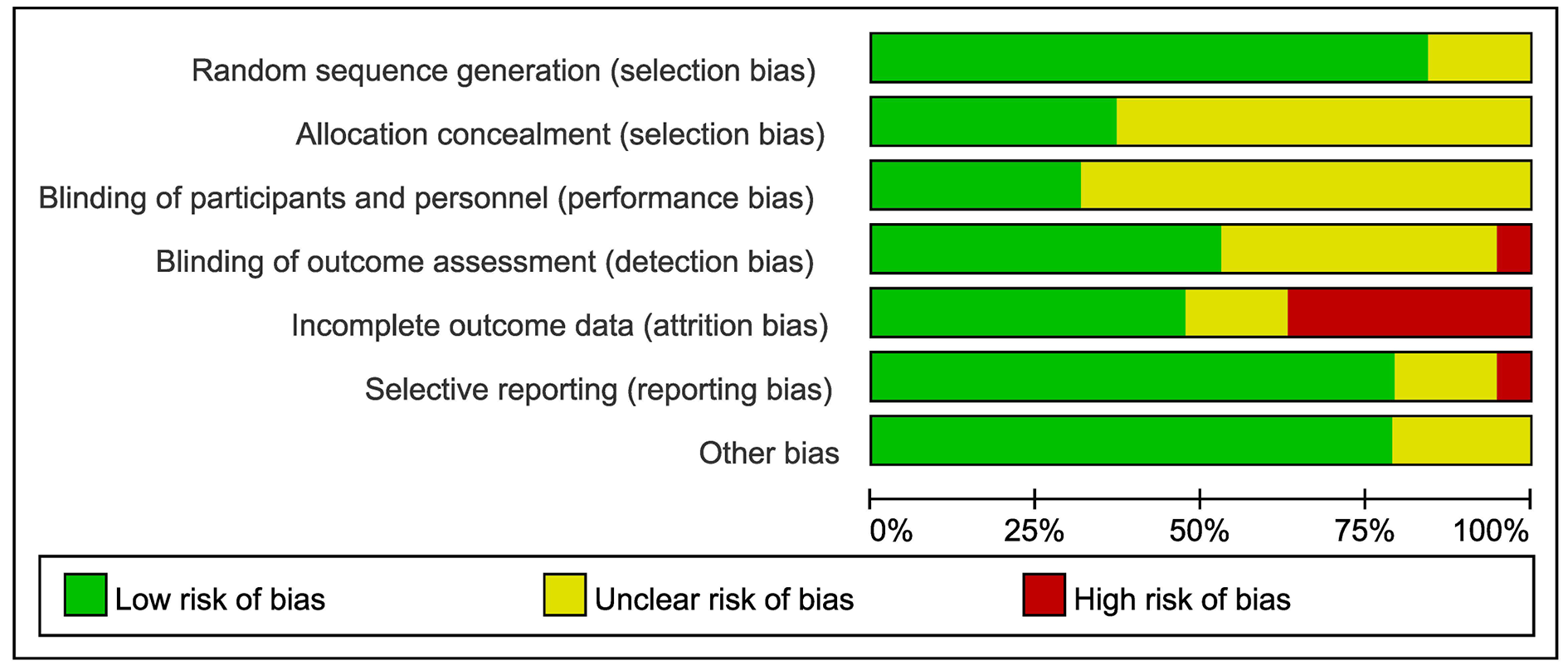

Supplement: Supplementary file 1 — Supplementary Material 1 [file 10072_2024_7918_MOESM1_ESM.tif]

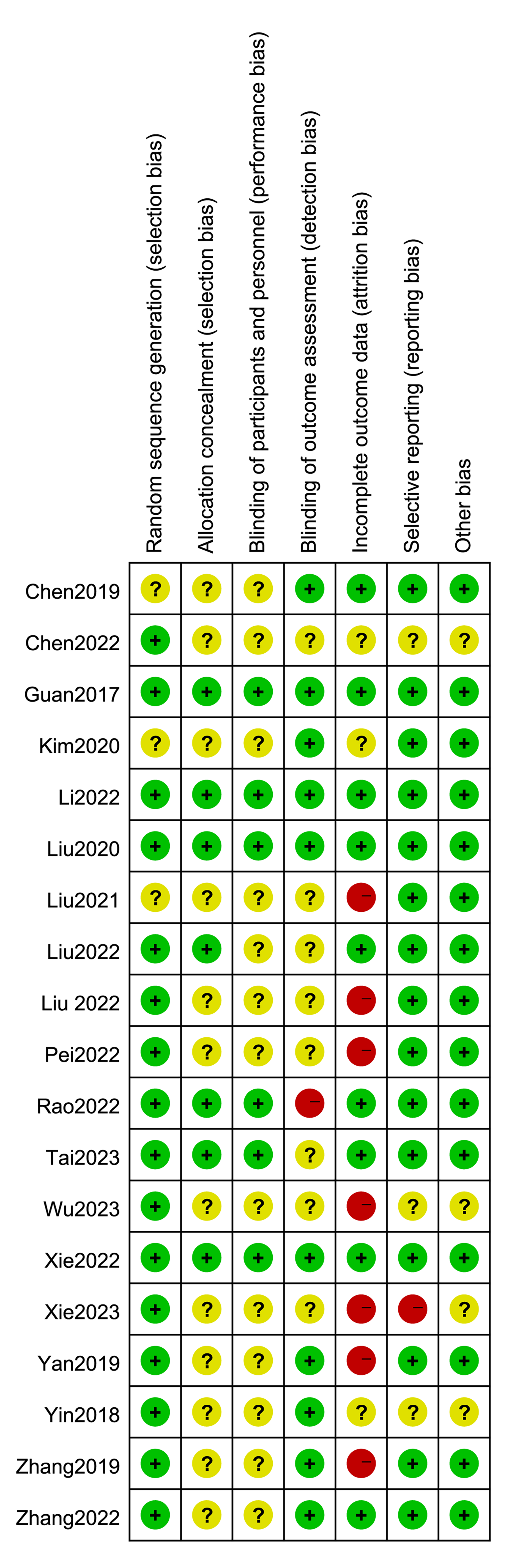

Supplement: Supplementary file 2 — Supplementary Material 2 [file 10072_2024_7918_MOESM2_ESM.tif]

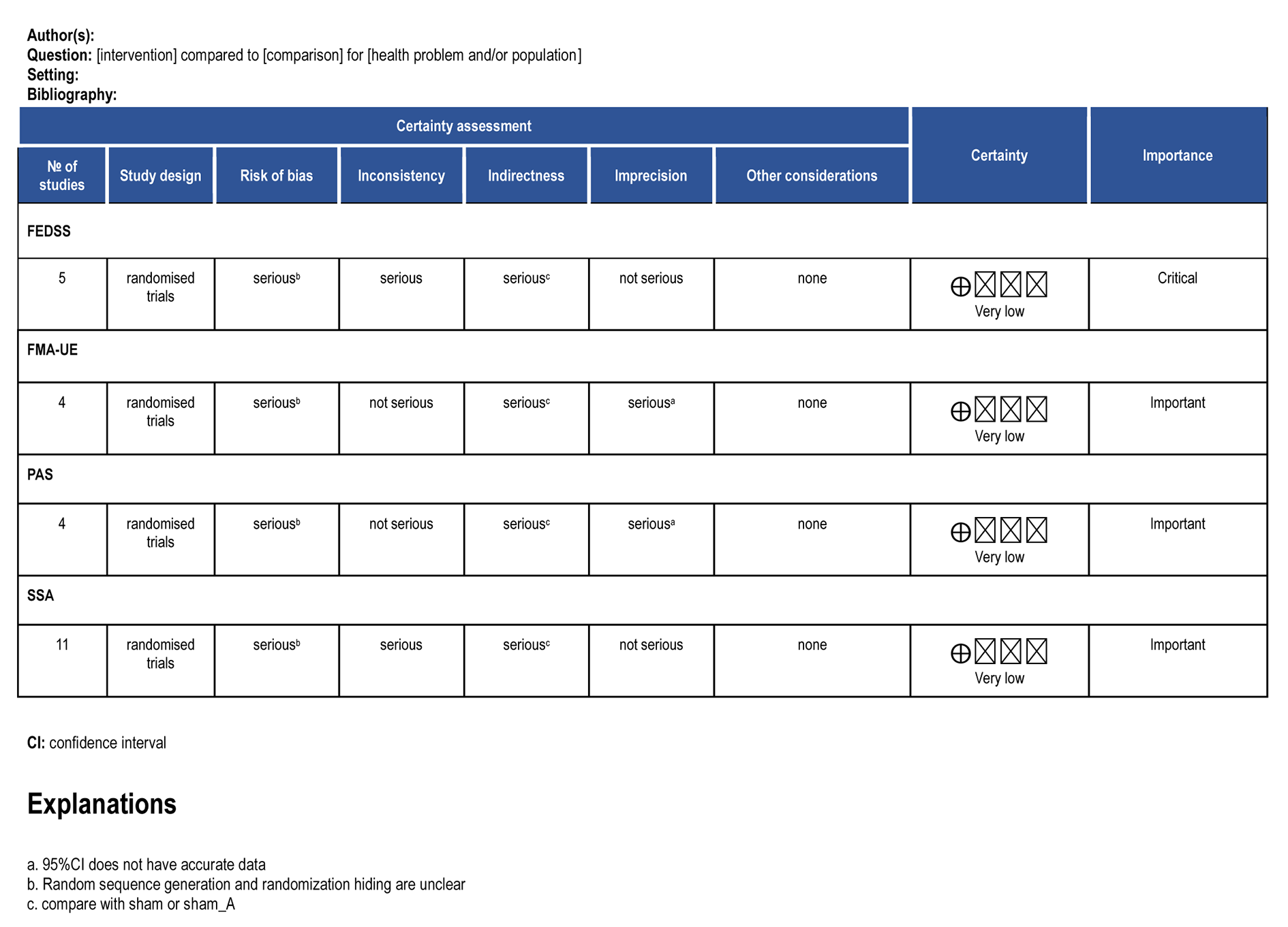

Supplement: Supplementary file 3 — Supplementary Material 3 [file 10072_2024_7918_MOESM3_ESM.tif]

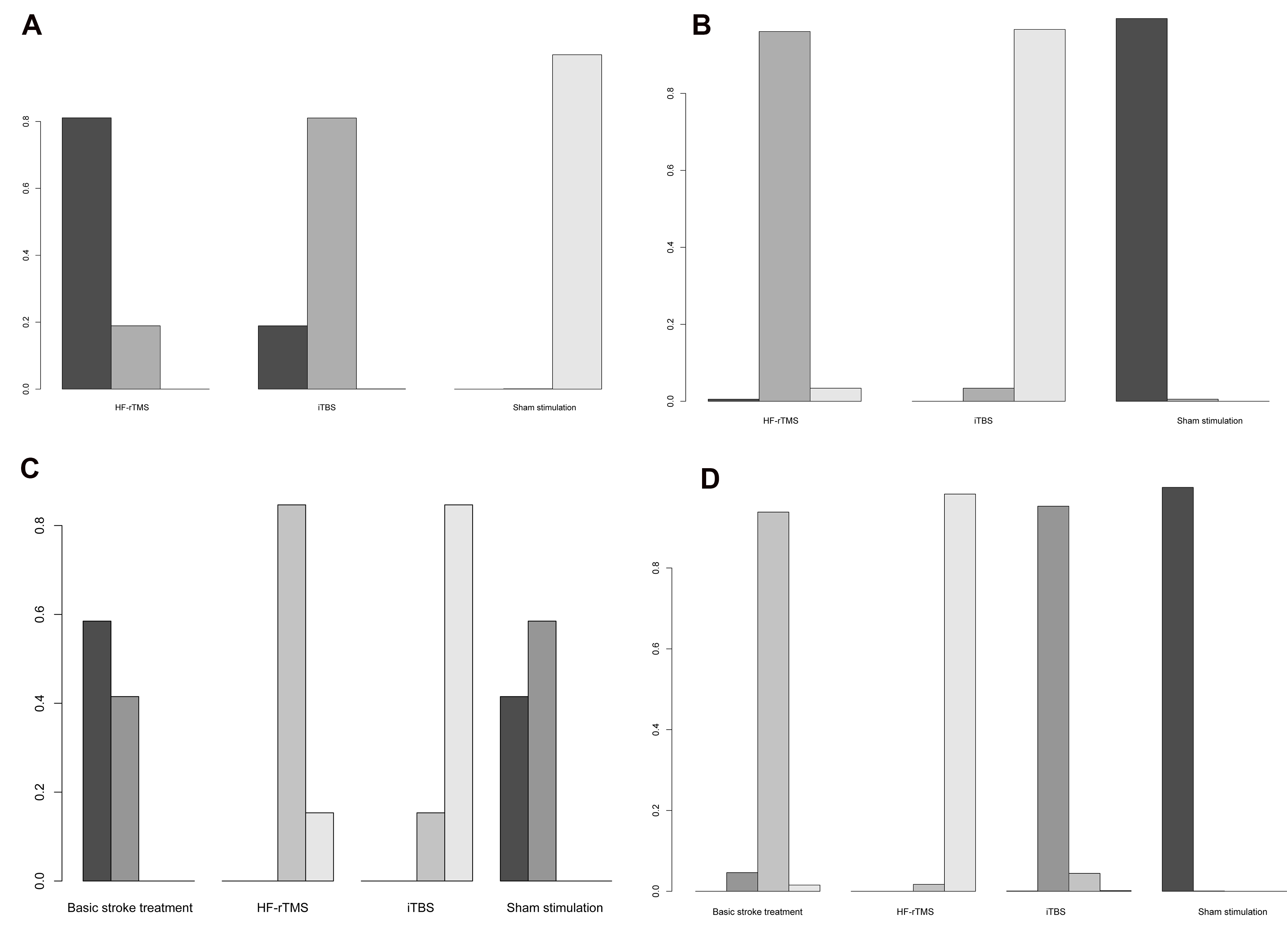

Supplement: Supplementary file 4 — Supplementary Material 4 [file 10072_2024_7918_MOESM4_ESM.tif]
