# Supplementary material for: Intermittent theta burst stimulation vs. high-frequency repetitive transcranial magnetic stimulation for post-stroke dysfunction: a Bayesian model-based network meta-analysis of RCTs
Source: Neurol Sci. 2024 Dec 21;46(4):1525–39. doi: 10.1007/s10072-024-07918-6 (PMC11919949; doi:10.1007/s10072-024-07918-6)
Supplement: Supplementary file 5 — Supplementary Material 5 [file 10072_2024_7918_MOESM5_ESM.docx]

Table Supplementary1 The search strategies of PubMed

| **Items** | **Content** |
| --- | --- |
| #1 | Stroke*  Cerebrovascular Accident*  CVA *(Cerebrovascular Accident)  Cerebrovascular Apoplexy  Apoplexy, Cerebrovascular  Vascular Accident*, Brain  Brain Vascular Accident*  Cerebrovascular Stroke*  Stroke*, Cerebrovascular  Apoplexy  Cerebral Stroke*  Stroke*, Cerebral  Stroke*, Acute  Acute Stroke*  Cerebrovascular Accident*, Acute  Acute Cerebrovascular Accident* |
| #2 | Theta burst stimulation (θ burst stimulation/TBS)  Theta-Burst Stimulation  Theta Burst Stimulation  Theta Burst  theta burst stimulation |
| #3 | transcranial magnetic stimulation*(TMS  Magnetic Stimulation*, Transcranial  Stimulation*, Transcranial Magnetic  Transcranial Magnetic Stimulation, Single Pulse  Transcranial Magnetic Stimulation, Paired Pulse  Transcranial Magnetic Stimulation, Repetitive  repetitive transcranial magnetic stimulation(rTMS) |
| Search formula | ((((((((((transcranial magnetic stimulation*[Title/Abstract]) OR (TMS[Title/Abstract])) OR (Magnetic Stimulation*, Transcranial[Title/Abstract])) OR (Stimulation*, Transcranial Magnetic[Title/Abstract])) OR (Transcranial Magnetic Stimulation, Single Pulse[Title/Abstract])) OR (Transcranial Magnetic Stimulation, Paired Pulse[Title/Abstract])) OR (Transcranial Magnetic Stimulation, Repetitive[Title/Abstract])) OR (repetitive transcranial magnetic stimulation[Title/Abstract])) OR (rTMS[Title/Abstract])) OR (((((((Theta burst stimulus[Title/Abstract]) OR (θ burst stimulation[Title/Abstract])) OR (TBS[Title/Abstract])) OR (Theta-Burst Stimulation[Title/Abstract])) OR (Theta Burst Stimulation[Title/Abstract])) OR (Theta Burst[Title/Abstract])) OR (theta burst stimulation[Title/Abstract]))) AND ((((((((((((((((Stroke*[Title/Abstract]) OR (Cerebrovascular Accident*[Title/Abstract])) OR (CVA *(Cerebrovascular Accident[Title/Abstract]))) OR (Cerebrovascular Apoplexy[Title/Abstract])) OR (Apoplexy, Cerebrovascular[Title/Abstract])) OR (Vascular Accident*, Brain[Title/Abstract])) OR (Brain Vascular Accident*[Title/Abstract])) OR (Cerebrovascular Stroke*[Title/Abstract])) OR (Stroke*, Cerebrovascular[Title/Abstract])) OR (Apoplexy[Title/Abstract])) OR (Cerebral Stroke*[Title/Abstract])) OR (Stroke*, Cerebral[Title/Abstract])) OR (Stroke*, Acute[Title/Abstract])) OR (Acute Stroke*[Title/Abstract])) OR (Cerebrovascular Accident*, Acute[Title/Abstract])) OR (Acute Cerebrovascular Accident*[Title/Abstract])) |
