# Supplementary material for: Intermittent theta burst stimulation vs. high-frequency repetitive transcranial magnetic stimulation for post-stroke dysfunction: a Bayesian model-based network meta-analysis of RCTs
Source: Neurol Sci. 2024 Dec 21;46(4):1525–39. doi: 10.1007/s10072-024-07918-6 (PMC11919949; doi:10.1007/s10072-024-07918-6)
Supplement: Supplementary file 6 — Supplementary Material 6 [file 10072_2024_7918_MOESM6_ESM.docx]

Table Supplementary2 Ranking of pairwise comparison of different treatments for post-stroke dysfunction

| **FMA-UE** |  |  |  |  |
| --- | --- | --- | --- | --- |
|  | HF-rTMS | iTBS | Sham stimulation |  |
| HF-rTMS | HF-rTMS | -1.64 (-4.45, 1.19) | -3.80 (-6.29, -1.31) |  |
| iTBS | 1.64 (-1.19, 4.45) | iTBS | -2.16 (-3.49, -0.85) |  |
| Sham stimulation | 3.80 (1.31, 6.29) | 2.16 (0.85, 3.49) | Sham stimulation |  |
| **SSA** ^a^ |  |  |  |  |
|  | Basic stroke treatment | HF-rTMS | iTBS | Sham stimulation |
| Basic stroke treatment | Basic stroke treatment | -3.10 (-3.91, -2.29) | -3.87 (-5.52, -2.22) | -0.49 (-2.00, 1.02) |
| HF-rTMS | 3.10 (2.29, 3.91) | HF-rTMS | -0.77 (-2.20, 0.67) | 2.61 (1.34, 3.88) |
| iTBS | 3.87 (2.22, 5.52) | 0.77 (-0.67, 2.20) | iTBS | 3.38 (2.39, 4.36) |
| Sham stimulation | 0.49 (-1.02, 2) | -2.61 (-3.88, -1.34) | -3.38 (-4.36, -2.39) | Sham stimulation |
| **SSA** ^b^ |  |  |  |  |
|  | Basic stroke treatment | HF-rTMS | iTBS | Sham stimulation |
| Basic stroke treatment | Basic stroke treatment | -2.74 (-3.59, -1.89) | -3.04 (-6.43, 0.35) | -0.89 (-3.30, 1.52) |
| HF-rTMS | 2.74 (1.89, 3.59) | HF-rTMS | -0.30 (-3.57, 2.99) | 1.85 (-0.40, 4.11) |
| iTBS | 3.04 (-0.35, 6.43) | 0.30 (-2.99, 3.57) | iTBS | 2.15 (-0.23, 4.52) |
| Sham stimulation | 0.89 (-1.52, 3.30) | -1.85 (-4.11, 0.4) | -2.15 (-4.52, 0.23) | Sham stimulation |
| **SSA** ^c^ |  |  |  |  |
|  | Basic stroke treatment | HF-rTMS | iTBS | Sham stimulation |
| Basic stroke treatment | Basic stroke treatment | -2.50 (-3.20, -1.80) | -2.81 (-6.16, 0.52) | -0.66 (-3.03, 1.71) |
| HF-rTMS | 2.50 (1.80, 3.20) | HF-rTMS | -0.31 (-3.57, 2.95) | 1.8 (-0.42, 4.11) |
| iTBS | 2.81 (-0.52, 6.16) | 0.31 (-2.95, 3.57) | iTBS | 2.15 (-0.22, 4.51) |
| Sham stimulation | 0.66 (-1.71, 3.03) | -1.84 (-4.11, 0.42) | -2.15 (-4.51, 0.22) | Sham stimulation |
| **SSA** ^d^ |  |  |  |  |
|  | HF-rTMS | iTBS |  |  |
| HF-rTMS | HF-rTMS | -0.75 (-2.49, 1.01) |  |  |
| iTBS | 0.75 (-1.01, 2.49) | iTBS |  |  |
| Sham stimulation | -3.14 (-4.73, -1.54) | -3.88 (-5.32, -2.44) |  |  |

iTBS, Intermittent theta-burst stimulation; HF-rTMS, High-frequency transcranial repeated magnetic stimulation; FMA-UE, Fugl-Meyer assessment for upper extremities; SSA, Standard swallowing function evaluation scale; FEDSS, Fiberoptic endoscopic dysphagia severity scale; PAS, Penetration/aspiration scale.

FMA-UE is a sensitivity analysis that excluded studies with different stimulated sites. ^a^: Sensitivity analysis that excluded studies with the longest duration of intervention; b: Sensitivity analysis that excluded studies with different stimulation (stimulated on the affected side group); c: Subgroup analysis of studies stimulated on the affected side; d: Subgroup analysis of studies stimulated on both sides.
